# Environmental Tobacco Smoke in Occupational Settings: Effect and Susceptibility Biomarkers in Workers From Lisbon Restaurants and Bars

**DOI:** 10.3389/fpubh.2021.674142

**Published:** 2021-06-04

**Authors:** Nádia Vital, Susana Antunes, Henriqueta Louro, Fátima Vaz, Tânia Simões, Deborah Penque, Maria João Silva

**Affiliations:** ^1^Department of Human Genetics, Instituto Nacional de Saúde Doutor Ricardo Jorge, Lisbon, Portugal; ^2^Centre for Toxicogenomics and Human Health (ToxOmics), NOVA Medical School/Faculdade de Ciências Médicas, Universidade Nova de Lisboa, Lisbon, Portugal

**Keywords:** second-hand smoke, occupational exposure, human biomonitoring, genotoxicity, challenge assay

## Abstract

Environmental tobacco smoke (ETS) has been recognized as a major health hazard by environmental and public health authorities worldwide. In Portugal, smoke-free laws are in force for some years, banning smoking in most indoor public spaces. However, in hospitality venues such as restaurants and bars, owners can still choose between a total smoke-free policy or a partial smoking restriction with designated smoking areas, if adequate reinforced ventilation systems are implemented. Despite that, a previous study showed that workers remained continuously exposed to higher ETS pollution in Lisbon restaurants and bars where smoking was still allowed, comparatively to total smoke-free venues. This was assessed by measurements of indoor PM_2.5_ and urinary cotinine, a biomarkers of tobacco smoke exposure, demonstrating that partial smoking restrictions do not effectively protect workers from ETS. The aim of the present work was to characterize effect and susceptibility biomarkers in non-smokers from those hospitality venues occupationally exposed to ETS comparatively to non-exposed ones. A group of smokers was also included for comparison. The sister chromatid exchange (SCE), micronucleus (MN) and comet assays in whole peripheral blood lymphocytes (PBLs) and the micronucleus assay in exfoliated buccal cells, were used as biomarkers of genotoxicity. Furthermore, a comet assay after *ex vivo* challenge of leukocytes with an alkylating agent, ethyl methanesulfonate (EMS), was used to analyze the repair capacity of those cells. Genetic polymorphisms in genes associated with metabolism and DNA repair were also included. The results showed no clear association between occupational exposure to ETS and the induction of genotoxicity. Interestingly, the leukocytes from non-smoking ETS-exposed individuals displayed lower DNA damage levels in response to the *ex vivo* EMS challenge, in comparison to those from non-exposed workers, suggesting a possible adaptive response. The contribution of individual susceptibility to the effect biomarkers studied was unclear, deserving further investigation.

## Introduction

Environmental tobacco smoke (ETS), also known as second-hand smoke (SHS), passive smoke or involuntary smoke is a widespread indoor pollutant of significant public health concern, and a major risk factor for lung cancer and other diseases (1–5). The mutagenic and carcinogenic effects of tobacco smoke have been clearly demonstrated (1, 2, 6, 7) and its adverse effects are not limited to smokers, but affect also environmentally and occupationally exposed non-smokers, since it is present in all places where smoking takes place (2). ETS is a significant source of a complex mixture of hundreds of hazardous substances comprising the smoke emitted from the burning tip of a cigarette (or other burned tobacco product) between puffs (sidestream smoke, SM), the smoke exhaled by the smoker (mainstream smoke, MS), and also the compounds diffused through the cigarette paper wrapper (1, 2, 8). ETS is classified as carcinogenic to humans (group 1) by the International Agency for Research on Cancer (1, 2), based on a clear evidence of a causal association between exposure of non-smokers and cancer. Because of its rapid dilution and dispersion into the indoor environment, the concentrations of individual ETS constituents can vary with time and environmental conditions (1, 2), and currently there is no safe level of exposure to ETS (9). While social ETS exposure can be controllable (an individual may avoid to be in places where smokers are present), employees, however, have little or no influence over ETS and may be exposed for a large part of their working day (10). Occupational exposure to ETS was associated with an increase of 16–19% in the risk of lung cancer among never-smokers (1). Comprehensive smoke-free laws offer the only effective means of eliminating the risks associated with ETS (9). In fact, to protect people from ETS, since 2005, smoke-free policies have been expanding worldwide covering indoor public places and workplaces, including hospitality venues (9, 11, 12) and overall have been highly effective in reducing the exposure to constituents of ETS (5, 11, 13, 14), as well as decreasing ETS-related diseases (12, 15), particularly when complete smoke-free ban is applied. Nonetheless, ETS remains a common indoor air pollutant, especially in indoor leisure areas including restaurants, bars, nightclubs and casinos (9, 16–18). In Portugal, the law that prohibits smoking in most indoor public spaces and workplaces was introduced in 2008, but in some cases, such as restaurants and bars, partial smoking restrictions are applied, and smoking is still allowed in separate designated smoking areas if adequate reinforced ventilation systems are implemented. Whether the health of the ETS-exposed workers is affected remained an open question. A previous work by Pacheco et al. (17) showed that ETS indoor pollution, estimated by the concentration of particulate matter (PM_2.5_), was consistently higher in restaurants where smoking was still allowed, comparatively to non-smoking restaurants and canteens (total smoke-free). In addition, the measurement of a biomarker of tobacco smoke exposure, i.e., cotinine, a metabolite of nicotine, confirmed the employees' exposure to ETS. Although all workers exhibited normal lung function, a proteomics approach identified differentially expressed proteins in the plasma of those ETS-exposed non-smoking workers, suggestive of alterations that may precede the first symptoms of tobacco-related diseases (19).

It is acknowledged that many substances contained in cigarette smoke are genotoxic and therefore genotoxicity biomarkers are good biomarkers to assess early effects from exposure to tobacco smoke (8, 20, 21), including ETS. The genotoxicity of ETS exposure has been addressed in few *in-vitro, in-vivo* and biomonitoring studies (8). In humans, environmental room exposure studies using fresh diluted sidestream smoke as a surrogate to estimate the effect of ETS exposure showed a slightly increased urinary mutagenicity (22) and DNA damage (23) in non-smoking voluntaries. Importantly, some studies reported the presence of DNA adducts (24), protein adducts (25–28) and urinary metabolites of carcinogens (29–31) after ETS exposure. On the other hand, biomonitoring studies evidencing the genotoxic effects of ETS on humans are scarce. While for chromosome instability results were predominantly negative (32–36), for the induction of DNA strand breaks, both positive (37, 38) and negative (39) results are described, although in the majority of those studies cotinine measurements confirmed the ETS exposure. A more evident genotoxic effect of ETS exposure appears to happen in children. A marginally significant increases of sister chromatid exchange (27), micronucleus (25, 40) and DNA damage measured with the comet assay (41–43) was reported. In the occupational settings, the impact of ETS on the genotoxicity biomarkers remains to be clarified.

The aim of the present work was to characterize the local and systemic genotoxic effects induced by occupational exposure to ETS in non-smoking workers from Lisbon restaurants and bars and to assess whether the genetic susceptibility could influence the observed effects. The sister chromatid exchange, micronucleus and comet assays in PBLs and the micronucleus assay in exfoliated buccal cells, were used to assess DNA and chromosome damage in ETS-exposed workers comparatively to non-exposed workers from the previously characterized hospitality venues (17, 19). A group of smokers working in the same venues was also included for comparison. In addition, the capacity of leukocytes to repair DNA lesions was estimated by the comet assay following their *ex vivo* exposure to an alkylating agent, ethyl methanesulfonate. Because several studies have evidenced the influence of genetic polymorphisms in genes encoding for metabolizing enzymes or DNA repair proteins on smoking-associated biomarkers, genotoxicity biomarkers and cancer predisposition (20, 44–50), some relevant susceptibility biomarkers were also studied. These included polymorphisms in metabolism (*GSTP1*^*105*^, *GSTM1*, and *GSTT1*) and DNA repair (*hOGG1*^*326*^*, XRCC1*^*194*^, *XRCC1*^*399*^*, XRCC3*^*241*^, *NBN*^*185*^*, PARP1*^*762*^*)* genes.

## Materials and Methods

### Workplace Characterization and Study Population

Among leisure establishments in Lisbon, restaurants and bars/discotheques were preselected based on a convenience sample. Accordingly, 58 main venues' owners were invited to participate in the study by letter and personal approach. After detailed information of project objectives, 25 agreed to participate in the study. Venues were classified as smoke-free (SFre), smoking (Sre) and mixed restaurants and bars with both smoking (Sro) and non-smoking rooms (NSro), as previously described by Pacheco et al. (17). In addition, four public institutions canteens (Cant) where smoking was not allowed were also included. ETS was assessed by monitoring the level of indoor air contaminants, namely, particulate matter (PM_2.5_), CO and CO_2_ in all venues and a full description of the methods and results obtained can be found elsewhere (17, 19).

To estimate the adequate sample size of the study groups, a power analysis was performed based on the frequency of micronuclei (MN) in lymphocytes, a sensitive biomarker of an early biological effect. Based on published and our own data from control groups, the mean frequency of MN was expected to be 7.0/1,000 cells and the SD = 3.0. An 80% power is generally considered as acceptable (51). To obtain a two-tailed *p*-value of 0.05 and a difference between the exposed and control groups that corresponds to a 25% higher mean level of MN among the exposed, a minimum of 49 subjects would be needed in each group; if the difference increases to 30%, 33 subjects would be needed. Despite the estimates made and the invitation to participate to a larger number of employees, only 97 accepted to provide blood and buccal cells samples for genotoxic assessment.

Ethics approval for this study was secured from Instituto Nacional de Saúde Dr. Ricardo Jorge (INSA) ethics committee, Lisbon. Each potential participant was informed about the procedures and the objectives of the study and those who accepted to participate provided written informed consent for the collection and utilization of biological specimens. During the medical surveillance phase of the study, each subject was interviewed to evaluate clinical history, demographic and lifestyle information, particularly about smoking habits (including amount, frequency, and duration of smoking) or self-reported exposure to ETS at home. According to the inclusion criteria, healthy subjects with more than 18 years, who worked in the above referred hospitality venues for more than 9 h per week and for at least 1 month at the current workplace were included in the study. Excluded were the individuals submitted to X-rays, blood transfusion or surgery between 0 and 2 months before the study and those who suffer or had suffered from cancer. Thus, from the 97 volunteers preselected, 81 were included in effect and susceptibility biomarkers analysis. Detailed contextual data can be found in Pacheco et al. (19).

Workers were separated into three study groups according to smoking status and ETS occupational exposure as follow: a group of non-smoking workers (NSW, *n* = 62) that was subdivided according to ETS exposure on the workplace into the ETS-exposed group (E, *n* = 29) including workers from Sre or mixed restaurants and bars and the non-exposed group (NE, *n* = 33) including workers from SFre and Cant; a group of smoking workers (SW, *n* = 19) containing workers from SFre and from Sre or mixed venues (17).

Human exposure to ETS and confirmation of smoking habits had been previously assessed by urinary cotinine levels, allowing to discriminate between smokers and non-smokers and between non-smokers exposed and not exposed to ETS. A full description of the methods and results obtained can be found elsewhere (17, 19).

### Biological Samples Collection

Following the interview and medical examination, biological samples were collected and coded to ensure their anonymization. For effect biomarkers characterization, peripheral blood and buccal epithelial cells samples were collected from each subject by medical personnel. Two mL of peripheral blood was collected by venipuncture into heparin-coated tubes and were processed within 2–3 h for SCE, MN and comet assays. Each subject was then asked to rinse the mouth twice with water and buccal epithelial cells were collected by gently scraping the oral mucosa of the inner lining of both cheeks with a plastic spatula. For genotyping of genetic polymorphisms, 2 mL of peripheral blood was collected into EDTA tubes, also by venipuncture.

### The Alkaline Comet Assay in Peripheral Blood Lymphocytes

The alkaline version of the comet assay was used to evaluate DNA damage in PBLs from each subject and was carried out as described elsewhere (52), with some modifications. Briefly, 20 μL of whole blood was added to a 1 ml phosphate-buffered saline (PBS, Gibco-Invitrogen, Carlsbad, CA). Cells were pelleted and 40 μL were embedded in 0.7% low-melting point agarose (Sigma, St. Louis, MO) and then dropped onto microscope slides pre-coated with 1% agarose (Amersham Biosciences, Uppsala, Sweden) and covered with a coverslip for about 10 min, at 4°C. Simultaneously, to test the response of PBLs to an *ex vivo* challenge, 20 μL of whole blood was added to 1 ml PBS (Gibco-Invitrogen) and exposed to 32 mM of ethyl methanesulfonate (EMS, Sigma), incubated at 37°C for 30 min, and equally processed. After gel solidification, coverslips were removed and slides were immersed in freshly prepared ice-cold lysis solution (2.5 M NaCl, 100 mM Na_2_EDTA.H_2_0, 10 mM Tris HCL, NaOH, pH 10) with 10% Dimethyl Sulfoxide (DMSO, Sigma) and 1% Triton X-100 (Sigma), for 1 h, at 4°C, in the dark. After lysis, slides were placed on a horizontal electrophoresis tank in an ice bath, immersed in alkaline electrophoresis buffer (300 mM NaOH, 1 mM Na2EDTA.2H2O, pH>13) in the dark, for 20 min, to allow DNA unwinding. Electrophoresis was then conducted at 25 V (~0.74 V/cm, 300 mA), at 4°C for 20 min. Then, slides were rinsed with the neutralization buffer (0.4 M Tris–HCl, pH 7.5), stained with 125 μg/mL ethidium bromide (Sigma), covered with a coverslip, and kept in a dark, moist chamber. Two slides were prepared for each subject and a “blind” scorer examined 50 randomly selected cells from each slide (100 cells/subject) using a 200× magnification in a Axioplan2 imaging epifluorescence microscope (Carl Zeiss Microscopy, Göttingen, Germany) with an image analysis system (Comet Imager 2.2 Software, MetaSystems, Altlussheim, Germany). The mean percentage of DNA in the nucleoids tail (tail DNA,%) and the tail length (TL) were calculated for each worker.

### Micronucleus Analyses in Peripheral Blood Lymphocytes

The Cytokinesis-blocked micronucleus assay (CBMN) was carried out as described elsewhere (53) with minor modifications. Briefly, whole blood samples (0.5 mL) from each subject were cultured in 4.5 mL RPMI-1640 medium with L- Glutamate (Gibco-Invitrogen) supplemented with Fetal Bovine Serum (25%, Sigma), phytohemaglutinin (2.5%, Gibco-Invitrogen), Penicillin-Streptomycin (1.5% Gibco-Invitrogen) and sodium heparin (0.5%, B. Braun Medical, Germany). Duplicate cultures from each subject were incubated at 37°C, for 68 h. Cytokinesis was blocked at 44 h of incubation by adding 5 μg/mL of cytochalasin B (Sigma-Aldrich, St. Louis, MO). After the 68 h of incubation, cells were harvested by treatment with a hypotonic solution (0.1 M KCl), at 37°C, followed by fixation (methanol: acetic acid, 3:1). Cells were immediately dropped onto microscope slides using cytocentrifugation, air-dried and stained with 4% Giemsa (Merck, Darmstadt, Germany) in pH 6.8 phosphate buffer. MN were blindly scored, under a bright field microscope (Axioskop 2 Plus, Zeiss, Germany) with a 400× magnification, and identified according to published criteria (54). From each subject at least one thousand binucleated cells (BC) with well-preserved cytoplasm (500 per replicate culture) were analyzed and the frequency of micronucleated binucleated cells per 1000 binucleated cells (MNBC/1000 BC) was calculated and represented as the mean number of MNBC/1000 BC ± SD. The proportion of mono- (MC), bi- (BC) or multinucleate cells (MTC) was determined in a total of 1,000 cells and the cytokinesis-block proliferation index (CBPI) was calculated as follows (55):

CBPI = (MC + 2BC + 3MTC)/Total Cells.

### Sister-Chromatid Exchange Analyses in Peripheral Blood Lymphocytes

For the SCE analysis in PBLs, cultures were established in duplicate as described previously (56), with minor modifications. One mL of whole blood were added to 9 mL RPMI-1640 culture medium supplemented as described above for the CBMN. Bromodeoxyuridine (BrdU, Sigma) was added to a final concentration of 10 μg/mL and incubated at 37°C for 56 h, in the dark. Cultures were treated with 0.1 mg/mL colcemid (Gibco-Invitrogen), 1 h prior to harvesting. Cells were processed through hypotonic treatment (0.075 M KCl) and fixation with methanol:acetic acid (3:1). Slides were prepared, air-dried and stained using the fluorescence plus Giemsa method (56, 57) for differential sister chromatids staining. For each subject, SCEs were analyzed in 50 second-division metaphases from two cultures, on coded slides, to determine the number of SCE per cell, and mean and standard deviation of the SCE counts per cell were calculated. The number of high frequency cells (HFCs) for each subject was determined as the proportion of metaphases whose SCE frequency exceeds the 95th percentile of the SCE distribution in the NE group, which was defined as those with a count of 14 or more SCEs.

### Micronucleus Analysis in Buccal Exfoliated Epithelial Cells

Buccal exfoliated epithelial cells were smeared onto slides, air-dried and fixed in 80% cold methanol, for 20 min (58). Slides were stained according to Feulgen's technique (59) without cytoplasm counterstain. Two thousand cells were scored on two slides (one from each cheek) from each individual (1000 cells per slide) based on published criteria (58, 59). Only cells containing an intact nucleus that was neither clumped nor overlapping were included in the analysis. The frequencies of micronucleated cells (MNC) and nuclear buds (NBUD) per 1000 cells were determined for each subject and represented as the mean number of MNC/1000 cells ± SD and NBUD/1000 cells ± SD, respectively.

### Genetic Polymorphisms in Metabolism and DNA Repair Genes

Genomic DNA was isolated from whole blood samples of the workers with the MagNA Pure LC DNA Isolation Kit (Roche Applied Science, Germany) following the manufacturer's instructions. DNA samples were stored at −20°C until analysis. Genetic polymorphisms in metabolism (*GSTP1*^*105*^, *GSTM1*, and *GSTT1*) and DNA repair (*hOGG1*^*326*^*, XRCC1*^*194*^, *XRCC1*^*399*^*, XRCC3*^*241*^, *NBN*^*185*^, and *PARP1*^*762*^) genes were analyzed using PCR-based assays, according to published methods with minor modifications. *GSTT1* and *GSTM1* genotype analysis was performed in the same reaction, in a multiplex PCR (60). DNA amplification by PCR with specific primers flanking the polymorphism, followed by enzymatic restriction and fragments' analysis by gel electrophoresis, i.e., the PCR-RFLP method, was used to characterize the following polymorphisms: *GSTP1*^*105*^ (61), *hOGG1*^*326*^ (62)*, XRCC1*^*194*^(63), *XRCC1*^399^(63)*, XRCC3*^*241*^(64), *NBN*^*185*^(65), and *PARP1*^*762*^ (66). The details of the primers, restriction enzymes, and PCR conditions are described in the Supplementary Table 1.

### Statistical Analysis

All statistical analyses were conduct using the IBM SPSS 17.0 for Windows statistical package. The level of significance considered was *p* < 0.05. The distribution of variables in total population and divided by groups was compared with the normal distribution by means of the Kolmogorov–Smirnov test. The studied variables MNBC/1000 BC cells, CBPI, MNC/1000 cells, NBUD/1000 Cells, SCE frequencies,% tail DNA, and TL departed significantly from normality and therefore non-parametric tests were applied. Chi-square test was applied to compare the frequency of MNBC/1000 BC and the frequency of MNC/1000 cells or NBUD/1000 cells in buccal epithelial cells between exposure groups. The frequencies of SCEs, HFCs, CPBI,% tail DNA and TL from each group were compared using the non-parametric Mann-Whitney U-test. The relationship between the biomarkers of early biological effects (MN and SCE frequencies, % tail DNA) and the duration of exposure to cigarette smoke (active and passive), cigarette consumption (number of cigarettes per day), cotinine concentration and age was explored by Spearman's correlation analysis. The same analysis was also used to explore correlations between the several effect biomarkers analyzed. The effect of gender on the cytogenetic parameters or in the genetic polymorphism was assessed by the Mann–Whitney *U*-test. Regarding the genetic polymorphisms, deviation from Hardy–Weinberg (HW) equilibrium was assessed with the Chi-Square-test. Statistical analysis using Pearson Chi-Square (2-sided), or two-sided Fisher's exact test were applied to assess differences between studied groups concerning allele distributions. To assess the influence of the genotype on each effect biomarker, non-parametric tests (Kruskal–Wallis or Mann–Whitney *U*-test) were applied. Two types of comparisons were made: the influence of the gene variants on the biomarker level within each exposure group and inter-group comparison according to each allelic variant. Due to the low number of homozygous variant carriers of *XRCC1*^*194*^, *XRCC1*^*399*^*, PARP1*^*762*^, and *XRCC3*^*241*^, all subjects harboring variant alleles (homozygous and heterozygous) were pooled together.

## Results

### Characteristics of the Study Population

General characteristics of the studied groups are described in Table 1. The studied population consisted of 81 workers, the majority being males (74%). The mean age was 45.2 ± 12.2 years for the NE group, 37.2 ± 10.8 years for the E group and 39.1 ± 11.1 years for SW. Mean age in the NE group was significantly higher than in E group (*p* = 0.008, unpaired *t*-test). Most employees stated to work at least 40 h per week (NE: 48.9 h ± 12.3; E: 39.1 ± 9.7 h; SW: 47.5 ± 12.6 h). Mean h per week in the E group was significantly lower than in NE group (*p* < 0.0001, Mann–Whitney *U*-test) and in SW group (*p* = 0.0024, Mann–Whitney *U*-test). The average months in the current job was higher in NE group (155.6 ± 151.4) in comparison with the E group (74.9 ± 91.2) and SW group (67.6 ± 54.3). Regarding smokers (SW), the average number of cigarettes smoked per day was 16.5 ± 7.5 (range 3–30), and the mean number of years as a smoker was 22.9 ± 10.9 (range 3–49). Most workers declared not being exposed to ETS out of the work, namely at home, and have no other professional activity where they could be occupationally exposed to ETS. Biological monitoring of ETS exposure was assessed by measurement of the urinary cotinine concentration, a metabolite of nicotine, using gas chromatography–mass spectrometry, as previously reported (17). As expected, the cotinine level was significantly increased in SW comparatively to NSW (*p* < 0.0001, Mann–Whitney *U*-test). The cotinine values obtained for non-smokers fall in the range considered involuntary exposure (17, 67). Among them, the mean cotinine level measured in the E group (7.98 ± 7.26 ng*/*mL) was significantly higher (*p* = 0.0005, Mann–Whitney *U*-test) than in the NE workers group (2.23 ± 4.31 ng*/*mL, the majority being below the level of quantification).

**Table 1:** Characteristics of the study population.

	**Smoking workers**	**Non-smoking workers**	
**Variables**	**(SW)**	**(NSW)**	
		**NE**	**E**
Number of workers	19	33	29
**Age (years)**			
Mean ± SD	39.1 ± 11.1	45.2 ± 12.2^a^	37.2 ± 10.8
Range	18–63	19–66	24–57
**Gender (%)**			
Female	6 (38.6)	11 (33.3)	4 (13.8)
Male	13 (68.4)	22 (66.7)	25 (86.2)
**Smoking habits**			
No. of cigarettes per day (Mean ± SD)	16.5 ± 7.5	–	–
Range	3–30	–	–
No. of years of smoking (Mean ± SD)	22.9 ± 10.9	–	–
Range	3–49	–	–
Other ETS exposure^*^ **(%)**			
Yes	4 (21.1)	3 (9.1)	7 (24.1)
No	12 (63.2)	30 (90.9)	17 (58.6)
No data	3 (15.8)	–	5 (17.2)
Biomarker of exposure^**^ **(ng/ml)**			
Cotinine concentration (Mean ± SD)	1598.3 ± 806.9	2.2 ± 4.3	7.9 ± 7.3^b^
Range	237.0–3125.0	1–19.0	1–28.0
**Working time characterization**			
Months in the current job (Mean ± SD)	67.6 ± 54.3	155.6 ± 151.4	74.9 ± 91.2
Range	3–180	2–468	1–408
Hour in a week of service (Mean ± SD)	47.5 ± 12.6	48.9 ± 12.3	39.1 ± 9.7^c^
Range	40–90	14–66	14–70

a *Mean NE significantly higher than E (p = 0.008, unpaired t**-**test);*

b *Mean E significantly higher than NE (p = 0.0005, Mann-Whitney U-test);*

c *Mean E significantly lower than NE (p < 0.0001) and SW (p < 0.01) with MannWhitney U-test;*

* *Exposure outside the workplace in the study, at home;*

** *Methodology described in Pacheco et al. (17); Cotinine average concentrations below the detection limit of the assay were assumed to be 0.1 ng/ml for analysis purpose (quantification limit is 5 ng/ml); SD, standard deviation*.

### Biomarkers of Early Biological Effects

The results of the cytogenetic and DNA damage effect biomarkers studied are presented in Tables 2, 3. In peripheral blood lymphocytes, no significant differences in the mean frequency of SCE/cell and in the level of HFCs were observed between the ETS-exposed (E) and NE groups. On the other hand, when considering the effect of smoking, a significantly higher percentage of HFCs was found in SW, comparatively to NE (*p* = 0.003, Mann–Whitney *U*-test) or to ETS-exposed groups (*p* = 0.016, Mann–Whitney *U*-test), although these differences were not detected when similar comparisons were made using the mean frequencies of SCEs (Table 2). The mean frequency of MNBC per 1000 BC was significantly different in the E as compared to the NE group (*p* = 0.004, Chi-square test) (Table 2). Unexpectedly, non-smoking workers exposed to ETS showed a 27.3% reduction in the frequency of MNBC in PBLs, as compared to non-smoking NE workers. Also, the SW group presented a significantly higher frequency of MNBC as compared with the ETS-exposed group (*p* = 0.001, Chi-square test), but no significant difference was detected between the group of SW and the NE group (*p* = 0.53, Fisher's Exact Test), both displaying a similar frequency of MNBC.

**Table 2:** Results of the cytogenetic effect biomarkers.

	**Smoking workers** **(SW)**		**Non-smoking workers** **(NSW)**				
			**NE**		**E**		***p*-value, test** **(NE *vs*. E)**
	***n***	**Mean ± SD**	***n***	**Mean ± SD**	***n***	**Mean ± SD**	
**Lymphocytes**							
SCEs	19	9.01 ± 1.64	33	8.06 ± 0.92	29	7.97 ± 1.42	0.472, Mann–Whitney
HFCs	19	14.00 ± 9.48^a^	33	6.79 ± 4.77	29	8.00 ± 7.54	0.892, Mann–Whitney
MNBC/1000 BC	19	6.79 ± 4.09	32	6.22 ± 3.27	29	4.52 ± 2.71^b^	0.004, Chi-square test
CBPI	19	1.56 ± 0.14	32	1.59 ± 0.16	29	1.61 ± 0.16	0.767, Mann–Whitney
**Buccal exfoliated cells**							
MNC/1000 cells	19	0.48 ± 0.59	32	0.49 ± 0.80	29	0.69 ± 0.90	0.306, Chi-square test
NBUD/1000 cells	19	0.31 ± 0.40^b^	32	0.85 ± 0.83	29	1.1 ± 0.83	0.318, Chi-square test

*SD, standard deviation; SCE, sister chromatid exchange; HFC, high frequency cells; MNBC, micronucleated binucleated cells; BC, binucleated cells; CBPI, Cytokinesis-blocked proliferation index; MNC, micronucleated cells; NBUD, nuclear buds;*

a *significantly different from NE (p = 0.003; Mann–Whitney U-test) and from E (p = 0.001; Mann–Whitney U-test);*

b *significantly different from SW (p = 0.001; Chi-square test);*

c *significantly different from both NE and E (p < 0.001, Chi-square test)*.

**Table 3:** Results of the comet assay in leukocytes (basal) and of the comet-based challenge assay.

	**Smoker workers** **(SW)**		**Non-smoker workers** **(NSW)**				
			**NE**		**E**		***p*-value**
	***n***	**Mean ± SD**	***n***	**Mean ± SD**	***n***	**Mean ± SD**	**(NE *vs*. E)**
**Basal**							
Tail DNA (%)	17	2.94 ± 0.94	32	2.93 ± 0.70	27	3.24 ± 1.34	0.738
Tail length (μm)	17	3.30 ± 1.64	32	3.13 ± 0.80	27	3.00 ± 0.90	0.523
**EMS challenge**							
Tail DNA (%)	17	35.46 ± 7.48^*^	32	36.67 ± 10.93	27	26.89 ± 6.95	0.0001
Tail length (μm)	17	35.70 ± 6.83^*^	32	36.02 ± 8.09	27	29.18 ± 5.90	0.0001

*SD, standard deviation; EMS, Ethyl Methanesulfonate;*

* *Significantly different from E groups (p < 0.01, Mann–Whitney U-test)*.

**Figure 1:**
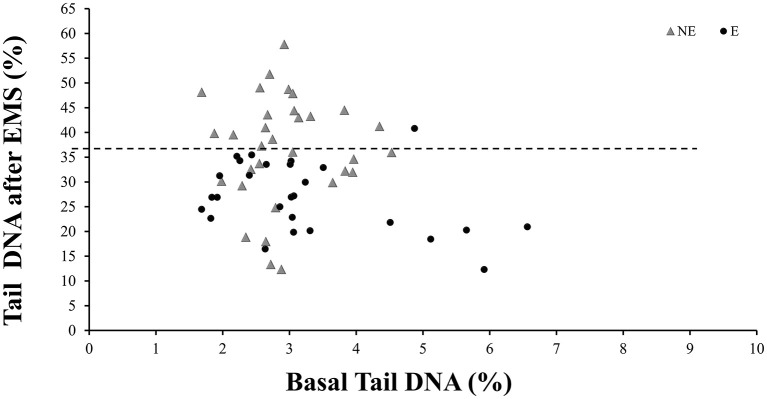
Distribution of the tail DNA from non-exposed (NE) or Exposed (E) workers with and without the *in vitro* challenge with Ethyl Methanesulfonate (EMS). Each data point corresponds to one worker. Dashed line represents the mean value of DNA in tail for the NE, after challenge with EMS.

As to the level of DNA damage assessed by the comet assay, no differences in the percentage of DNA in tail or in the tail length were observed neither between the E and NE groups nor between SW and NSW groups (Table 3). In respect to the *ex vivo* challenge assay with EMS, the data show that both parameters, tail DNA and TL, were significantly different in ETS-exposed comparatively to the NE group (*p* < 0.001, Mann–Whitney *U*-test). The level of EMS-induced DNA damage was 26.6% lower in the E group, as compared to the NE group of workers. The individual values of tail DNA for each worker, from E or NE groups, is shown in Figure 1. After the *ex vivo* EMS challenge assay, the distribution of the data points from the E workers is always under a threshold of 36%, while NE individuals show a wider range of DNA damage induction, up to 57% (Figure 1). When comparing the SW with the ETS-exposed group, a significant difference was also observed (*p* < 0.01, Mann–Whitney *U*-test), with the E group presenting the lowest values of tail DNA and TL in challenged lymphocytes; no difference was observed between the SW and the NE groups.

In Buccal exfoliated cells there were no significant differences between the E and NE groups, neither in respect to the frequency of MNC/1000 cells nor in NBUD/1000 cells. Considering smoking, the SW displayed the lowest NBUD frequencies and the comparison between the SW and NSW showed a significant difference in the frequency of NBUD (*p* = 0.0004, Chi-square test). A positive correlation was found between MNBC/1000 BC in PBLs and MNC/1000 cells (*p* = 0.003, *r* = 0.527, Spearman's correlation) or NBUD/1000 cells (*p* = 0.0038, *r* = 0.5199, Spearman's correlation) in buccal cells in the E group, which was not seen in other groups.

The impact of potential confounding factors was analyzed in respect to each of the effect biomarkers considering the total number of individuals studied, or after stratification by exposure. In this sense, data for each biomarker were separately analyzed according to gender, but no significant differences were observed, in spite women presented a slightly increased mean of SCEs when compared to males (9.33 ± 1.48 *vs*.7.75 ± 1.31, respectively; *p* = 0.071, Mann–Whitney *U*-test) and a slightly lower level of DNA damage as assessed by the percentage of DNA in tail (2.18 ± 0.31 *vs*. 3.38 ± 1.35, respectively; *p* = 0.076, Mann–Whitney U-test), only in the E group. None of the biomarkers was impacted by the age of the individuals. Regarding the smoking habits, there was no influence of the number of cigarettes smoked per day or the number of years of smoking in the biomarkers analyzed. Also, no relationship was found between the parameters that characterize the working time and the effect biomarkers. Furthermore, there was no correlation between the exposure biomarker (urinary cotinine concentration) and each of the effect biomarker, when considering the whole group of individuals or after their stratification according to exposure, i.e., E, NE, and SW groups.

### Biomarkers of Susceptibility

The distribution of the genotype frequencies relative to the metabolism and DNA repair genes in the studied groups is presented in Table 4. The allelic frequencies of *GSTP1*^*105*^, *hOGG1*^*326*^*, XRCC1*^*194*^, *XRCC1*^*399*^*, XRCC3*^*241*^, *NBN* ^*185*^, and *PARP1*^*762*^ follow the Hardy-Weinberg conditions (*p* < 0.05, Chi-square test), except the *GSTP1*^*105*^genotype on the ETS-exposed group. In the study population, considering the polymorphisms in the *hOGG1*^*326*^*, XRCC1*^*194*^, and *PARP1*^*762*^ genes, the prevalent allele was the common allele (+/+). In addition, when considering the *GSTT1* gene, the wild-type allele was prevalent in the study population; the E group presented a lower prevalence of the null genotype (17.24%) comparatively to the NE (39.39%). For the *GSTM1* gene both genotypes were similarly present (Table 4). The distribution of the common and variant alleles between the NE and E groups did not show significant differences for any of the polymorphism analyzed except for *NBN* ^*185*^ (*p* = 0.047, Fisher exact test), where the E group presented a lower prevalence of the wild-type genotype. No significant differences were observed between SW and E or NE groups for all studied polymorphisms, although the *GSTP1 Ile/Ile* genotype was more prevalent in the E group (62.07%) than in the SW (31.58%).

**Table 4:** Frequency of metabolism and DNA repair genotypes in the studied groups.

**Genes**	**Genotypes**	**All (%)**	**Smoker workers** **(SW) (%)**	**Non-smoker workers** **(NSW)**		***p*-value**
				**NE (%)**	**E (%)**	**(NE *vs*. E)**
*GSTP1^*105*^*	*Ile/Ile*	41(50.6)	6 (31.58)	17 (51.52)	18 (62.07)	0.679^a^
	*Ile/Val*	29 (35.8)	11 (57.89)	11 (33.33)	7 (24.14)	
	*Val/Val*	11 (13.6)	2 (10.53)	5 (15.15)	4 (13.79)	
	FA^*^	0.31	0.39	0.32	0.26	
*GSTM1*	*Present*	41 (50.6)	7 (36.84)	17 (51.52)	17 (58.62)	0.617^b^
	*Absent*	40 (49.4)	12 (63.16)	16 (48.48)	12 (41.38)	
*GSTT1*	*Present*	58 (71.6)	14 (73.68)	20 (60.61)	24 (82.76)	0.091^b^
	*Absent*	23 (28.4)	5 (26.32)	13(39.39)	5 (17.24)	
*XRCC1^*194*^*	*Arg/Arg*	66 (81.5)	18 (94.74)	25 (75.76)	23 (79.31)	0.771^b^
	*Arg/Trp*	15 (18.5)	1 (5.26)	8 (24.24)	6 (20.69)	
	*Trp/Trp*	0 (0.0)	0 (0.0)	0 (0.0)	0 (0.0)	
	FA^*^	0.09	0.03	0.12	0.1	
*XRCC1^*399*^*	*Arg/Arg*	40 (49.4)	10 (52.63)	14 (42.42)	16 (55.17)	0.559^a^
	*Arg/Gln*	35 (43.2)	9 (47.37)	15 (45.45)	11 (37.93)	
	*Gln/Gln*	6 (7.4)	0 (0.0)	4 (12.12)	2 (6.90)	
	FA^*^	0.29	0.24	0.35	0.26	
*XRCC3^*241*^*	*Thr/Thr*	33 (40.7)	10 (52.63)	11 (33.33)	12 (41.38)	0.356^a^
	*Thr/Met*	36 (44.4)	8 (42.11)	14 (42.42)	14 (48.28)	
	*Met/Met*	12 (14.8)	1 (5.26)	8 (24.24)	3 (10.34)	
	FA^*^	0.37	0.26	0.45	0.34	
*hOGG1^*326*^*	*Ser/Ser*	57 (70.4)	12 (63.16)	27 (81.82)	18 (62.07)	0.096^b^
	*Ser/Cys*	21 (25.9)	7 (36.84)	6 (18.18)	8 (27.59)	
	*Cys/Cys*	3 (3.7)	0 (0.0)	0 (0.0)	3 (10.34)	
	FA^*^	0.17	0.18	0.09	0.24	
*NBN^*185*^*	*Glu/Glu*	40 (49.4)	10 (52.63)	20 (60.61)	10 (34.48)	0.047^b^
	*Glu/Gln*	33 (40.7)	7 (36.84)	10 (30.30)	16 (55.17)	
	*Gln/Gln*	8 (9.9)	2 (10.53)	3 (9.09)	3 (10.34)	
	FA^*^	0.30	0.29	0.24	0.38	
*PARP1^*762*^*	*Val/Val*	54 (69.2)	12 (70.59)	20 (60.61)	22 (75.86)	0.170^b^
	*Val/Ala*	21 (26.9)	5 (29.41)	11 (33.33)	5 (17.24)	
	*Ala/Ala*	3 (3.9)	0 (0.0)	2 (6.06)	1 (3.45)	
	FA^*^	0.17	0.15	0.23	0.13	

* *FA - Frequency of the variant allele for each polymorphism, in the total population and in each studied group. The frequency of the variant allele was calculated considering the heterozygous plus homozygous individuals having the variant allele;*

a *Statistical analysis using Pearson Chi-Square (2-sided);*

b *Statistical analysis using Fisherexact test (2-sided)*.

### Influence of Genetic Susceptibility on Effect Biomarkers

To ascertain the potential influence of the metabolism and DNA repair genes genotype in the genotoxic outcomes, the measurements of chromosome and DNA damage in individuals carrying the common or the variant alleles were compared both in the total study population and in each study group (Tables 5, 6). When analyzing the total number of individuals studied, irrespectively of the exposure condition, none of the polymorphisms characterized significantly influenced the level of the effect biomarkers (*p* > 0.05, Mann-Whitney) (data not shown).

**Table 5:** Mean (± SD) frequencies of SCEs and MNBC in PBL in the studied groups stratified by genotypes.

		**SCEs**				**MNBC**			
**Genes**	**Genotypes**	**SW**	**NSW**		***p*-value^**2**^**	**SW**	**NSW**		***p*-value^**2**^**
			**NE**	**E**	**(NE *vs*. E)**		**NE**	**E**	**(NE *vs*. E)**
*GSTP1*^*105*^	*Ile/Ile*	8.87 ± 1.26	8.29 ± 0.94	7.87 ± 1.08	0.222	4.50 ± 2.35	6.29 ± 2.93	4.94 ± 3.32	0.139
	*Ile/Val*	8.53 ± 1.38	8.03 ± 0.70	8.25 ± 1.75	0.821	6.91 ± 3.96	6.60 ± 4.50	3.86 ± 1.07	0.257
	*Val/Val*	12.02 ± 0.85	7.38 ± 1.12	7.92 ± 2.38	0.806	13.0 ± 2.82	6.22 ± 3.27	3.75 ± 0.96	0.076
	*p-*value^1^	0.064	0.158	0.996		0.072	0.799	0.674	
*GSTM1*	*Present*	9.20 ± 2.00	8.14 ± 0.77	7.69 ± 1.34	0.241	8.14 ± 3.24^a^	6.50 ± 3.56	4.65 ± 3.40	0.053
	*Absent*	8.89 ± 1.48	7.98 ± 1.09	8.37 ± 1.45	0.763	6.00 ± 4.45	5.94 ± 3.04	4.33 ± 1.37	0.091
	*p*-value^2^	0.933	0.679	0.223		0.097	0.879	0.445	
*GSTT1*	*Present*	8.92 ± 1.63	8.11 ± 0.86	7.92 ± 1.54	0.346	6.60 ± 2.70	7.05 ± 3.56	4.63 ± 2.84	0.015
	*Absent*	9.25 ± 1.84	7.98 ± 1.05	8.22 ± 0.57	0.693	6.86 ± 4.57	5.00 ± 2.45	4.00 ± 2.12	0.481
	*p-*value^2^	0.711	0.754	0.386		0.963	0.096	0.907	
*XRCC1^*194*^*	*Arg/Arg*	9.10 ± 1.64^a^	7.97 ± 0.99	7.90 ± 1.47	0.536	7.11 ± 3.95^a^	6.32 ± 3.31	4.91 ± 2.81	0.084
	*Arg/Trp*	7.38	8.35 ± 0.63	8.23 ± 1.28	0.948	1.00	5.86 ± 3.34	3.00 ± 1.67	0.080
	*p-*value^2^	–	0.208	0.518		–	0.800	0.107	
*XRCC1^*399*^*	*Arg/Arg*	8.87 ± 1.34	8.00 ± 0.96	7.88 ± 1.30	0.574	7.10 ± 4.04^a^	5.79 ± 3.22	3.75 ± 1.48	0.080
	*Arg/Gln + Gln/Gln*	9.15 ± 1.99	8.11 ± 0.92	8.07 ± 1.59	0.759	6.44 ± 4.36	6.56 ± 3.87	5.45 ± 3.55	0.250
	*p-*value^2^	0.806	0.610	0.843		0.593	0.502	0.189	
*XRCC3^*241*^*	*Thr/Thr*	8.69 ± 1.50	8.23 ± 0.58	8.13 ± 1.7 1	0.558	6.80 ± 4.37	6.50 ± 3.21	4.75 ± 2.01	0.141
	*Thr/Met + Met/Met*	9.36 ± 1.80	7.98 ± 1.06	7.85 ± 1.21	0.671	6.78 ± 4.02^a^	6.09 ± 3.37	4.35 ± 3.16	0.041
	*p-*value^2^	0.414	0.349	0.690		0.773	0.622	0.252	
*hOGG1^*326*^*	*Ser/ser*	9.21 ± 1.46^b^	8.07 ± 0.95	8.32 ± 1.53	0.694	6.83 ± 4.13	6.59 ± 3.31	4.83 ± 2.83	0.024
	*Ser/Cys + Cys/Cys*	8.65 ± 1.98	8.01 ± 0.88	7.40 ± 1.03	0.174	6.71 ± 4.35	4.20 ± 2.39	4.00 ± 2.53	0.818
	*p-*value^2^	0.176	0.907	0.087		0.966	0.142	0.230	
*NBN^*185*^*	*Glu/Glu*	8.75 ± 1.62	8.20 ± 0.76	8.22 ± 1.01	0.660	5.70 ± 2.91	5.79 ± 3.41	3.70 ± 1.83	0.116
	*Glu/Gln*	9.21 ± 1.82^a^	7.82 ± 1.11	7.55 ± 1.46	0.580	8.43 ± 5.68	6.50 ± 3.34	5.19 ± 3.25	0.221
	*Gln/Gln*	9.57 ± 1.82	7.93 ± 1.44	9.35 ± 1.73	0.275	6.50 ± 0.71	8.00 ± 2.00	3.67 ± 0.58	0.046
	*p-*value^1^	0.697	0.399	0.184		0.623	0.364	0.405	
*PARP1*^*762*^	*Val/Val*	9.31 ± 1.76	8.03 ± 1.10	7.95 ± 1.36	0.571	7.42 ± 4.36	6.15 ± 3.23	5.09 ± 2.79	0.147
	*Val/Ala + Ala/Ala*	8.73 ± 1.61	8.11 ± 0.60	8.03 ± 1.85	0.335	5.00 ± 2.74	6.33 ± 3.47	3.00 ± 1.27	0.052
	*p-*value^2^	0.527	0.854	0.654		0.365	0.922	0.041	

1 *Statistical analysis using Kruskal–Wallis;*

2 *Statistical analysis using Mann–Whitney U-test;*

a *significantly different from E group, within same genotype (p < 0.05, Mann-Whitney U-test);*

b *significantly different from NE group, within same genotype (p < 0.05, Mann–Whitney U-test)*.

**Table 6:** Mean (± SD) values for basal and EMS tail DNA in the studied groups stratified by genotypes.

		**Basal tail DNA (%)**				**EMS tail DNA (%)**			
**Genes**	**Genotypes**	**SW**	**NSW**		***p-*value^**2**^**	**SW**	**NSW**		***p*-value^**2**^**
			**NE**	**E**	**(NE *vs*. E)**		**NE**	**E**	**(NE *vs*. E)**
*GSTP1*^*105*^	*Ile/Ile*	3.00 ± 0.43	2.86 ± 0.68	3.56 ± 1.44	0.163	35.20 ± 8.31	39.05 ± 10.14	26.65 ± 7.37	0.000
	*Ile/Val*	3.15 ± 1.06^a^	3.27 ± 0.75	2.26 ± 0.47	0.017	34.28 ± 7.23	35.11 ± 12.64	27.28 ± 6.25	0.104
	*Val/Val*	1.78 ± 0.13	2.53 ± 0.37	3.36 ± 1.23	0.142	42.06 ± 6.87	31.71 ± 9.76	27.30 ± 7.88	0.327
	*p-*value*^1^*	0.200	0.055	0.063		0.386	0.969	0.802	
*GSTM1*	*Present*	2.60 ± 0.88	2.80 ± 0.62	3.53 ± 1.45	0.132	34.61 ± 7.10^a^	34.29 ± 13.69	26.34 ± 7.40	0.09
	*Absent*	3.13 ± 0.96	3.07 ± 0.76	2.83 ± 1.08	0.236	35.93 ± 7.98^a^	39.05 ± 6.897	27.68 ± 6.50	0.001
	*p-*value*^2^*	0.763	0.407	0.103		0.763	0.522	0.622	
*GSTT1*	*Present*	3.15 ± 0.95	3.08 ± 0.64	3.27 ± 1.44	0.734	37.60 ± 7.53^a^	34.56 ± 11.91	26.19 ± 7.06	0.010
	*Absent*	2.45 ± 0.79	2.71 ± 0.74	3.12 ± 0.84	0.349	30.35 ± 4.62^b^	39.75 ± 8.88	29.96 ± 6.11	0.016
	*p-*value*^2^*	0.171	0.186	0.851		0.073	0.140	0.236	
*XRCC1^*194*^*	Arg/Arg	2.94 ± 0.94	2.90 ± 0.66	3.29 ± 1.37	0.609	35.56 ± 7.48^a^	37.18 ± 10.89	26.84 ± 7.25	0.001
	Arg/Trp	–	3.05 ± 0.87	3.05 ± 1.28	0.808	–	34.87 ± 11.77	27.10 ± 6.13	0.123
	*p-*value*^2^*	–	0.837	0.618		–	0.665	0.950	
*XRCC1^*399*^*	*Arg/Arg*	3.12 ± 0.28	2.95 ± 0.51	3.21 ± 1.03	0.533	35.58 ± 7.69^a^	37.32 ± 12.64	26.30 ± 6.23	0.011
	*Arg/Gln + Gln/Gln*	2.79 ± 1.28	2.92 ± 0.83	3.29 ± 1.74	0.893	35.37 ± 7.75	36.17 ± 9.75	27.74 ± 8.11	0.015
	*p-*value*^2^*	0.290	0.849	0.622		0.847	0.820	0.693	
*XRCC3^*241*^*	*Thr/Thr*	2.91 ± 0.69	3.03 ± 0.69	3.13 ± 1.17	0.725	34.30 ± 6.04	40.13 ± 11.88	28.82 ± 7.24	0.006
	*Thr/Met + Met/Met*	2.98 ± 1.16	2.89 ± 0.71	3.32 ± 1.46	0.515	36.50 ± 8.80^a^	35.10 ± 10.38	25.56 ± 6.64	0.004
	*p-*value*^2^*	0.441	0.684	0.805		0.770	0.155	0.278	
*hOGG1^*326*^*	Ser/ser	3.15 ± 1.01	2.88 ± 0.67	3.32 ± 1.43	0.66	33.93 ± 8,06	35.97 ± 11.17	28.56 ± 6.35	0.007
	Ser/Cys + Cys/Cys	2.66 ± 0.80	3.24 ± 0.84	3.09 ± 1.18	0.641	37.66 ± 6.50^a^	40.47 ± 9.72	23.55 ± 7.23	0.014
	*p-*value*^2^*	0.696	0.364	0.959		0.283	0.517	0.072	
*NBN^*185*^*	*Glu/Glu*	3.09 ± 1.19	2.98 ± 0.84	2.79 ± 1.19	0.337	34.83 ± 6.52	34.83 ± 11.2 4	28.00 ± 6.50	0.058
	*Glu/Gln*	2.84 ± 0.54	2.92 ± 0.48	3.65 ± 1.43	0.222	38.06 ± 9.18^a^	37.56 ± 9.98	25.41 ± 7.54	0.008
	*Gln/Gln*	2.60 ± 0.86	2.93 ± 0.70	2.57 ± 0.43	0.513	30.53 ± 6.39	45.35 ± 10.93	30.95 ± 3.58	0.050
	*p-*value*^1^*	0.796	0.796	0.163		0.454	0.388	0.290	
*PARP1*^*762*^	*Val/Val*	2.91 ± 0.61	2.85 ± 0.67	3.26 ± 1.34	0.45	36.29 ± 7.30^a^	37.83 ± 10.44	26.13 ± 7.05	0.000
	*Val/Ala + Ala/Ala*	3.07 ± 2.23	3.07 ± 0.75	3.15 ± 1.46	0.527	37.61 ± 8.75	34.73 ± 11.93	30.22 ± 5.95	0.399
	*p-*value*^2^*	0.564	0.613	0.755		0.773	0.276	0.190	

1 *Statistical analysis using Kruskal–Wallis;*

2 *Statistical analysis using Mann–Whitney U-test;*

a *significantly different from E group, within same genotype (p < 0.05, Mann-Whitney U-test);*

b *significantly different from NE group, within same genotype (p < 0.05, Mann–Whitney U-test)*.

Considering the *GSTP1, GSTM1*, and *GSTT1* polymorphisms, no significant differences amongst the possible genotypes were observed, within SW, NE or E groups, for the frequencies of SCEs, MNBC or DNA strand breaks (% tail DNA). It may be noted that, in the SW group, the frequencies of SCEs, HFCs (data not shown) and MNBC were higher in the subjects with the *GSTP1* variant allele (only two individuals), comparatively to those of the WT or heterozygous carriers. The E group maintained the overall trend of lower MNBC comparatively to both SW and NE group which was not influenced by the genotype. On the other hand, SW individuals with the *GSTM1* allele present, showed significantly increased MNBC when compared to E group, while *GSTT1* wild-type individuals from NE group presented increased MNBC when compared to E group (*P* = 0.015, Mann-Whitney *U*-test). In respect to the EMS-induced DNA damage, irrespective of the genotype, the already observed lower levels of DNA damage in the E group comparatively with the NE or SW group is maintained either for the *GSTP1, GSTM1*, or *GSTT1*. However, workers' stratification according to their genotype, lead to a lower statistical power, due to the small samples size. This is reflected in the *GSTP1* and *GSTM1*, where for *GSTP1* the difference between the ETS-exposed and NE groups only became significant for the subset of the WT allele carriers whereas for the *GSTM1* polymorphism significance was detected for the comparison between the null allele carriers. Interestingly, in the case of SW, in the absence of the *GSTT1*, a lower level of DNA damage was observed comparatively to *GSTT1* wild-type individuals, an effect that was opposite of the observed in both E and NE groups.

For the *XRCC1*^*194*^, *XRCC1*^*399*^ and *XRCC3*^*241*^, *hOGG1*^*326*^ and *NBN* ^*185*^ polymorphisms, no significant influence of the genotype was observed on the frequencies of SCEs, HFCs (data not shown), MNBC or tail DNA, within NE, E, or SW groups. In respect to *PARP1*^*762*^ polymorphism, a significant difference in the frequency of MNBC was observed within the E group (*p* = 0.041), with the variant allele carriers showing a lower frequency of micronucleated cells than the WT ones. Overall, the E group maintained the trend of lower MNBC and EMS-induced DNA damage comparatively to both SW and NE group.

## Discussion

Environmental tobacco smoke is a serious public health concern, recognized as one of the most common indoor pollutants worldwide. Many countries have already successfully implemented smoke-free laws for indoor public spaces and workplaces aimed at limiting exposure to ETS. In Portugal, since 2008, a partial smoke-free law is in place, allowing exceptions, as for example in the case of restaurants, bars or discotheques where smoking is allowed in smokers' designated areas if adequate reinforced ventilation systems are implemented. Thus, exposure to ETS still happens in some Portuguese restaurants and bars (16, 17, 19), meaning that workers remain at risk of ETS exposure. Since tobacco smoke contains a great variety of genotoxic/carcinogenic agents, this study aimed at characterizing the local and systemic biomarkers of genotoxic effects associated to occupational ETS exposure in a set of Lisbon restaurants and bars and the potential influence of genetic polymorphisms on those biomarkers. The quantification of employee's exposure to ETS and the self-reported smoking status was confirmed through urinary cotinine measurement (17).

In the present study, no effect could be ascribed to ETS exposure in relation to the basal level of DNA damage, as assessed by the comet assay in leukocytes. A similar negative result was obtained when comparing smokers to non-smokers, irrespectively of the ETS exposure. The effect of smoking on DNA damage has been thoroughly studied, mainly as a confounding factor in biomonitoring studies addressing exposure to other compounds (68, 69). Despite that, discrepant results have been reported in the literature, either describing a lack of association between smoking and DNA damage induction (the majority of studies), as reviewed elsewhere (68–70) or an increased DNA damage in smokers comparatively to non-smokers (37, 38, 71–74). Very few studies have been published in respect to the effect of ETS exposure on this biomarker and contradictory results have been reported. In accordance with our results, in peripheral blood lymphocytes of active and involuntary smoking pregnant women, no significant difference was observed between involuntary smokers and non-smokers, but smoking mothers exhibited a statistically significant increase in DNA damage comparatively to involuntary smokers (39). Moreover, newborns displayed results similar to those found for their mothers (39). An increase in DNA damage was reported in lymphocytes of white-collar involuntary smokers and smokers at workplace, comparatively to never smokers, although the mean value obtained for involuntary smokers was similar to ours (38). A similar observation was described in another study, in workers from an elevator manufacturing factory in China, potentially exposed to benzene, were passive smoking at home, but not at the workplace, was significantly associated with DNA damage (37). In children, a significant increase in DNA damage has also been reported after exposure to ETS (41–43). No influence of age or gender in the DNA damage was observed in our study either in the total population or in the studied groups, although women presented a slightly lower level of DNA damage, as assessed by the percentage of DNA in tail. The impact of age on DNA damage is a matter of controversy, with either positive or negative findings reported, which might depend of different factors such as life-style, descriptors or statistics used, as recently discussed (68). Regarding gender, the overall studies have demonstrated no or equivocal difference between men and women (68). Nevertheless, a study on the impact of ETS exposure in non-smoking workers from casinos and bars in Las Vegas, DNA-damage was significantly increased in a dose-dependent manner with ETS exposure in non-smoker men but not in women (10), in agreement with our finding.

Challenging lymphocytes *ex vivo* with a genotoxicant (e.g., EMS) and measuring induced primary DNA lesions with the comet assay is a functional assay that allows an indirect measurement of the DNA repair competence, which is critical to prevent permanent genetic instability (75, 76). In fact, it is well-known that abnormal DNA repair is a major cause and is mechanistically involved in the development of cancer (77, 78). In this study, blood cells exposure to a single EMS dose allowed the identification of a differential responses in the ETS-exposed group, which presented significantly lower levels of DNA damage, comparatively to the NE and to SW groups. This response suggests that leukocytes from involuntary smokers somehow managed better the EMS-induced alkylating lesions, promoting their rapid repair. In the study of Fracasso et al. (38), lymphocytes of active and non-smokers exposed to ETS challenged with an exogenous oxidative agent (i.e. H_2_O_2_-induced DNA damage) showed that never smokers not exposed to ETS, had the highest rates of repair of H_2_O_2_-induced breaks and that the lymphocytes of active smokers exhibited a consistent repair rate at the two administered doses (100 and 200 μM), slower that never smokers, possible due to the presence of high levels of DNA lesions, hardly or not at all repaired (38). However, the passive non-smokers displayed a reduced DNA repair efficacy comparatively to ex-smokers exposed to ETS or to active smokers. The reason for this distinct effect compared to our results, might be related to the challenge agent used. Vodicka et al. reported a higher irradiation-specific DNA repair rate among highly exposed workers in a rubber tire plant and in styrene-exposed lamination workers compared to unexposed or moderately exposed workers (79, 80). The apparent increase in DNA repair following a genotoxicant's acute exposure may reflect a general activation of the DNA repair machinery (79). A similar effect was observed following exposure to benzene (81). In our study, we hypothesize that cells from ETS-exposed individuals may display an adaptive response after EMS challenging due to the continuous low-level exposure to tobacco smoke that works like the conditioning dose. The concept of adaptive response is based on the observation that exposure of cells to a low conditioning genotoxic insult (e.g., radiation, bleomycin, mitomycin C, ethylnitrosurea) leads to their protection against a subsequent higher (challenge) dose of the same genotoxicant, an effect that may result from the upregulation of DNA repair functions (82). For example, there are several lines of evidence for an increase of O^6^-methylguanine-DNA methyltransferase (MGMT) activity in the normal tissue of smokers compared to non-smokers although these data awaits conclusive proofs (83). However, Au et al. using the challenge assay, showed that workers exposed to butadiene, pesticides and styrene, and residents exposed to uranium mining and milling waste were found to have significantly higher induction of chromosome aberrations than the respective matched-controls, supposedly due to impaired DNA repair capacity (77, 84).

Therefore, although in this study ETS exposure did not affect the basal level of DNA damage, there is a suggestion that it modulates the blood cells DNA repair response. It remains to be determined whether increased DNA repair capacity in exposed individuals is truly induced, and if so whether a threshold exists for this induction, and whether long-term exposure could exhaust the induction as suggested by Vodicka et al. (80). Furthermore, these findings suggest that ETS, activates a response mechanism that counteracts the negative effects ETS exerted on DNA and the preliminary investigation to try to identify such mechanisms using a proteomic-based approach is ongoing (19).

No increase in either the frequency of SCEs or HFC was observed in ETS-exposed workers (involuntary smokers), as compared to non-exposed workers. Our finding agrees with the negatives data reported in previous studies in workers exposed to ETS in restaurants (33) and in administrative companies (35). The latter studied 106 adult non-smokers divided into two groups according to whether they experienced high or low levels of exposure to ETS as determined from plasma cotinine levels. Nevertheless, in children, ETS exposure was associated with increased SCE (27). On the other hand, it is recognized that the measurement of SCEs in PBLs is a sensitive biomarker of exposure to cigarette smoke (32, 33, 85–92). The cigarette smoke effect was not observed in our study, mainly constituted by light smokers (amount of daily cigarette consumption: 16.47 ± 7.25; range 3–30). In that regard, a previous study in healthy individuals, showed no differences between light smokers and non-smokers, while the percentages of HFC were significantly higher in smokers than in non-smokers (86). Other studies also observed an effect of smoking in HFC but not in SCE frequency, although only in 1,3-butadiene-exposed workers (93). In our study, the proportion of HFCs was also significantly higher in SW comparatively to the whole non-smokers group and the same trend was observed after stratification into ETS-exposed and NE workers. HFCs are considered relevant to assess the genotoxicity of human chronic exposure to chemicals and have been identified as long-lived lymphocytes which accumulate persistent damage or as a subpopulation of lymphocytes with an increased sensitivity to chemicals (87, 94). Therefore, analyzing the percentage of HFC, as a “measure of SCE rate” may be more sensitive than the mean of SCE to detect effects due to chemical exposure, such as smoking, when an effect is not clearly detected by differences in mean SCE value (95). Our results concerning the influence of gender agree with published studies showing no association with the SCE frequency (87, 92, 96), although positive findings were also reported showing that women display a higher frequency of SCE comparatively to men (85). Also, no influence of age was observed in agreement with recent studies (85, 92).

Interestingly, a lower frequency of micronucleated cells was found in PBLs of involuntary smokers, comparatively to NE or to SW groups, whereas NE and SW displayed a similar frequency of MNBC. It is important to refer that the frequencies of MNBC obtained for NE and SW groups were within the range of our historical control values. Although this observation in the E group was unexpected, other studies have reported that light smokers (smoking <20 cigarettes per day) and former smokers displayed slightly reduced MN frequencies in comparison to non-smokers (21, 97). An explanation may rely on the fact that the most damaged cells may not survive the culture period in the CBMN assay or may not be able to divide and thus to express chromosome breaks or loss as MN in cultured lymphocytes (21, 97). Another hypothesis also pointed out by the same authors to justify the lower frequency of MN observed in the PBL of light-medium smokers when compared with non-smokers, is that a few cigarettes per day may stimulate an adaptive response, causing an apparent lowering in the MN frequency, and a continued exposure to mutagens/carcinogens may induce resistance to further DNA damage (21, 97). This might be the case in our study, since the observed lower frequency of MN in involuntary smokers is compatible with the hypothesized increased capacity of these workers to repair DNA strand breaks, following *ex vivo* blood cells exposure to EMS. Taken together, the results from both assays may suggest that continuous and repetitive exposure to low level of ETS stimulates a cell-protective response. However, it must stressed that the long-term health consequences from this continuous stimulation cannot be foreseen. A study addressing the effect of ETS exposure on children showed a 30% increase in the frequency of MN (25), although caution should be taken before making extrapolations to adults, since children show increased sensitivity to toxic substances when compared to adults due to differences in chemicals detoxification and excretion pathways (76, 98, 99). In this study, no induction of MNBC was observed in SW as compared to the total NSW population not stratified by ETS exposure. Although some authors reported an increase in the MN frequency in smokers, our results agree with those from a meta-analysis within the HUMN project, that showed that smokers do not exhibit an overall increased MN frequency when compared to non-smokers, which is normally higher in heavy smokers not occupationally exposed to genotoxic agents (21). This was also recently observed in other study (100). Although the effect of age and gender on the MN levels in lymphocytes is well-established, with women having higher levels of MN than men and with MN levels progressively increasing with age (97, 101, 102), that was not observed in our study, possibly due to the sample size and the relatively young age of the participants, mostly constituted by men.

The analysis of MN and NBUD in buccal cells, as a biomarker of local effect, did not detect differences in the number of MNC and NBUD between the E and NE groups, with both groups presenting MN frequencies similar to the average reported for healthy population (1–3 per 1,000 cells) (101–103). A positive correlation between MN in buccal cells and MN in whole blood lymphocytes was found in our study, but only for the E group. This observation agrees with a recent analysis showing that MN frequencies in exfoliated buccal cells correlate with those analyzed in peripheral lymphocytes and that both are valid biomarkers for increased cancer risk in humans (104, 105). However, a decreased level of NBUD was seen in the SW group comparatively to NSW, which might be related to a higher turnover of the oral mucosa cells of smokers. Neither age or gender influenced the level of MNC or NBUDs in our study. Also, within the HUMN(XL), in an analysis of a database of 5,424 subjects with buccal MN values obtained from 30 laboratories worldwide, no effect of gender was evident, while the trend for age was highly significant (102).

The remaining confounding factors, either related to smoking habits or working time did not influence the effect biomarkers analyzed. Furthermore, there was no correlation between the exposure biomarker (urinary cotinine concentration) and each of the biomarkers of effect, when considering the whole group of individuals or after their stratification according to exposure, i.e., E, NE, and SW groups.

Overall, the results of a set of biomarkers of early biological effects showed that ETS did not induce DNA or chromosome damage in blood cells from non-smoking exposed workers. Furthermore, following an *ex vivo* acute genotoxic stimulus, ETS-exposed individuals displayed a higher competence to repair DNA damage than unexposed individuals, which might be related with an adaptive response triggered by the prolonged exposure to a low level of tobacco smoke components.

Genetic susceptibility biomarkers, such as the inherited capacity for xenobiotic biotransformation and DNA damage repair, indicate individual differences that can modulate the response to genotoxic insults (46, 47). Thus, the association of polymorphisms in relevant genes with biological effects following exposure to environmental stressors represents a valuable tool for assessing the individual sensitivity to that exposure, and it may also influence the basal level of DNA or chromosome damage (47, 50). Therefore, in this study, the influence of genetic polymorphisms in genes associated with metabolism and DNA repair on several genotoxicity endpoints was analyzed. For most polymorphisms investigated, no influence was detected in relation to any of the genotoxicity biomarkers within workers from each of the studied groups, possibly due to small sample size. The E group maintained the overall trend of lower MNBC and EMS-induced DNA damage, comparatively to both SW and NE group, which was not influenced by the genotype. The only exception was *PARP1*^*762*^ polymorphism. PARP1 is an enzyme involved in the cellular response mechanisms to DNA damage. It participates in DNA base excision repair, single- and double-strand break repair pathways, that are active in the prevention of deletions/insertions induced by alkylating agents. *PARP1* polymorphism *Val762Ala* (rs1136410 T>C) may be associated with prostate cancer (106), esophageal squamous cell carcinoma (107), and breast cancer (108). Recent meta-analyses found a borderline significant association between *PARP1 Val762Ala* polymorphism and overall cancer risk, although after stratification by cancer types, the polymorphism could predispose to gastric cancer, thyroid cancer and cervical cancer, in an Asian population, but not in Caucasian and African populations (109, 110). Involuntary smoking was associated with an increased risk of breast cancer among both pre- and postmenopausal women, depending on the genotype of *PARP1*^*762*^ (111), while *Ala762Ala* (rs1136410 C/C) genotype was associated with an elevated risk of esophageal squamous cell carcinoma in smokers compared to T/T or T/C genotype (107, 112). In the present study, the *PARP1*^*762*^ homozygous wild-type genotype modulated the MNBC frequency, which was lower in variant carriers, within the E group. Considering the above-mentioned studies, it would be expected that the variant allele would rather be associated with increased MNBC frequency. When looking at EMS-induced DNA damage, the *PARP1*^*762*^ variant allele carriers presented an increase of DNA damage in E group comparatively to the wild-type carriers, although not significant, due to small number of variant allele carriers. It can be suggested that the partial suppression of PARP1 activity in the variant carriers might have led to an overload of genetic damage in cells, triggering cell death mechanisms and removal of most damaged cell, thereby masking a genotoxic effect. Besides, a decreased MNCB frequency was observed in the variant allele carriers from E group compared to NE, similarly to the slight effect observed in E workers with normal allele, thus suggesting a possible interaction between tobacco smoke exposure and the genotype, in E group, in the genotoxic outcome observed. According to recent meta-analyses, *GSTM1* and *GSTT1* deletion as been associated with lung cancer in overall population (113, 114), while no correlation was observed concerning *GSTP1*^*105*^, in overall population (115). Concerning DNA repair-genes, based on meta-analysis, *XRCC1*^*399*^ and *XRCC1*^*194*^ polymorphism were significantly associated with lung cancer risk in caucasions (116), and *NBS1*^*195*^ in Asians, but not caucasions (117). No relation with lung cancer was observed either in *XRCC*3^*241*^ and *hOGG1*^*326*^ genotype subjects (118–120). Overall, despite the influence of the studied polymorphisms in the genotoxic biomarkers was not sharp when considering the exposure groups stratification, some influence appears to exist that needs however, to be further investigated in larger groups of workers.

In the present study the results of genotoxicity biomarkers used to assess the early biological effects of ETS in non-smoking workers are presented. The most relevant effect detected in restaurant workers exposed to indoor tobacco smoke was a modified response to a genotoxic challenge, compatible with an adaptive response. It remains to be determined, however, whether the induction of this kind of response may have long term consequences to the health of those workers. The other effects biomarkers characterized in lymphocytes or oral mucosa cells did not detect significant changes in ETS-exposed comparatively to unexposed workers. Although the contribution of individual susceptibility to the outcomes of this exposure did not generate conclusive results, it deserves further attention. Further investigation is also needed to better understand the mechanisms underlying the possible adaptive response reported in this work and its implication to human health. Ongoing work will try to address the relationship between ETS exposure, biomarkers of effect and alterations of the proteome.

## Data Availability Statement

The raw data supporting the conclusions of this article will be made available by the authors, without undue reservation.

## Ethics Statement

The studies involving human participants were reviewed and approved by Comissão de ética do Instituto Nacional de Saúde Doutor Ricardo Jorge. The patients/participants provided their written informed consent to participate in this study.

## Author Contributions

NV, SA, and HL carried out the experiments and drafted the manuscript. MS and HL conceived the experimental strategy, supervised the work, participated in the data analysis and discussion, and in the manuscript writing and revision. TS and DP were the project coordinators and implemented the study logistics. FV was involved in workers recruitment, contextual data, and samples collection. All authors read and approved the final manuscript.

## Conflict of Interest

The authors declare that the research was conducted in the absence of any commercial or financial relationships that could be construed as a potential conflict of interest.
