# Supplementary material for: Environmental Tobacco Smoke in Occupational Settings: Effect and Susceptibility Biomarkers in Workers From Lisbon Restaurants and Bars
Source: Front Public Health. 2021 Jun 4;9:674142. doi: 10.3389/fpubh.2021.674142 (PMC8213454; doi:10.3389/fpubh.2021.674142)
Supplement: Supplementary file 1 [file Table_1.docx]

Supplementary Material

Environmental Tobacco Smoke in Occupational Settings: Effect and Susceptibility Biomarkers in Workers From Lisbon Restaurants and Bars

Supplementary Table 1. Primers, PCR conditions and enzymatic restriction conditions used.

| **Polymorphism** | **Primers** | **PCR conditions** | | | | | **Enzymatic restriction** | | **Ref.** |
| --- | --- | --- | --- | --- | --- | --- | --- | --- | --- |
|  |  | **PCR**  **cycling conditions** | | **PCR mix** | | **Product length (bp)** | **Enzymes** | **Product length (bp)** |  |
| *GSTP1^105^* | F - 5´ACCCCAGGGCTCTATGGGAA 3´  R - 5´TGAGGGCACAAGAAGCCCCT 3´ | 1x | 95ºC, 5min | MgCl_2_ Invitrogen^TM^ (mM) | 1.5 | 176 | *BsmAI* | WT: 176 | 61 |
|  |  | 35x | 94ºC, 45s  55ºC, 35s  72ºC, 1min | primer (pmol) | 12.5 |  |  |  |  |
|  |  |  |  | DNA (ng) | 50 |  |  | H: 176; 91; 85 |  |
|  |  |  |  | dNTPs (mM) | 0.2 |  |  |  |  |
|  |  | 1x | 72ºC, 10min | Buffer 10x Invitrogen^TM^ | 1x |  |  | HV:  91; 85 |  |
|  |  |  |  | Taq DNA Polymerase (U) | 1 |  |  |  |  |
| *Multiplex GSTT1*  *GSTM1* | *GSTT1*:  F - 5′TCACCGGATCATGGCCAGCA 3´  R - 5′TTCCTTACTGGTCCTCACATCTC 3′ | 1x | 94ºC, 5min. | MgCl_2_ Invitrogen^TM^ (mM) | 1.5 | *GSTT*1:  480 *GSTM1*:  215 *CYP1A1*:  459 | *na* | *na* | 60 |
|  |  | 35x | 94ºC, 2min  59ºC, 1min  72ºC, 1min | primer (pmol) | 30 |  |  |  |  |
|  | *GSTM*1:  F - 5′GTTGGGCTCAAATATACGGTGG 3′  R - 5′GAACTCCCTGAAAAGCTAAAGC 3′ |  |  | DNA (ng) | 50 |  |  |  |  |
|  |  |  |  | dNTPs (mM) | 0.2 |  |  |  |  |
|  | *CYP1A1* (control):  F - 5′GAACTGCCACTTCAGCTGTCT 3′  R - 5′CAGCTGCATTTGGAAGTGCTC 3′ | 1x | 72ºC, 10min | Buffer 10x Invitrogen^TM^ | 1x |  |  |  |  |
|  |  |  |  | Taq DNA Polymerase (U) | 2 |  |  |  |  |
| *XRCC1^194^* | F - 5´GCCAGGGCCCCTCCTTCAA 3´  R - 5´TACCCTCAGACCCACGAGT 3´ | 1x | 95ºC, 5min | MgCl_2_ Invitrogen^TM^ (mM) | 1.5 | 485 | *PvuII* | WT:485 | 63 |
|  |  | 35x | 95ºC, 30s  61ºC, 35s  72ºC, 45s | primer (pmol) | 12.5 |  |  |  |  |
|  |  |  |  | DNA (ng) | 50 |  |  | H: 485; 396; 89 |  |
|  |  |  |  | dNTPs (mM) | 0.25 |  |  |  |  |
|  |  | 1x | 72ºC, 10min | Buffer 10x Invitrogen^TM^ | 1x |  |  | HV: 396; 89 |  |
|  |  |  |  | Taq DNA Polymerase (U) | 0.5 |  |  |  |  |
| *XRCC1^399^* | F - 5´CAGTGGTGCTAACCTAAT 3´  R - 5´AGTAGTCTGCTGGCTCTGG 3´ | 1x | 95ºC, 5min | MgCl_2_ (mM) | 1.5 | 871 | *NciI* | WT: 461; 278; 132 | 63 |
|  |  | 35x | 95ºC, 45s  58ºC, 45s  72ºC, 1.2min | primer (pmol) | 12.5 |  |  |  |  |
|  |  |  |  | DNA (ng) | 50 |  |  | H: 593; 461; 278; 132 |  |
|  |  |  |  | dNTPs (mM) | 0.25 |  |  |  |  |
|  |  | 1x | 72ºC, 10min | Buffer 10x Invitrogen^TM^ | 1x |  |  | HV: 593; 278 |  |
|  |  |  |  | Taq DNA Polymerase (U) | 0.5 |  |  |  |  |
| *XRCC3^241^* | F - 5 GCCTGGTGGTCATCGACTC 3´  R -5´ACAGGGCTCTGGAAGGCACTG  CTCAGCTCACGCACC-3´ | 1x | 94ºC, 3min | MgCl2 (mM) | 1.5 | 136 | *Nco*I | WT: 136 | 64 |
|  |  | 35x | 95ºC, 1min  60ºC, 1min  72ºC, 1min | primer (pmol) | 12.5 |  |  |  |  |
|  |  |  |  | DNA (ng) | 100 |  |  | H:136;  39; 97 |  |
|  |  |  |  | dNTPs (mM) | 0.25 |  |  |  |  |
|  |  | 1x | 72ºC, 5min | Buffer 10x Invitrogen^TM^ | 1x |  |  | HV: 39; 97 |  |
|  |  |  |  | Taq DNA Polymerase (U) | 0.5 |  |  |  |  |
| *hOGG1^326^* | F - 5´CCCAACCCCAGTGGATTCTCATTG 3´  R - 5´GGTGCCCCATCTAGCCTTGCGGCCCTT 3´ | 1x | 94ºC, 4min | MgCl_2_ Invitrogen^TM^ (mM) | 2 | 213 | *Fnu4l* | WT: 213 | 62 |
|  |  | 35x | 94ºC, 30s  60ºC, 30s  72ºC, 1.5min | primer (pmol) | 12.5 |  |  |  |  |
|  |  |  |  | DNA (ng) | 100 |  |  | H: 213; 164; 49 |  |
|  |  |  |  | dNTPs (mM) | 0.25 |  |  |  |  |
|  |  | 1x | 72ºC, 5min | Buffer 10x Invitrogen^TM^ | 1x |  |  | HV: 164; 49 |  |
|  |  |  |  | Taq DNA Polymerase (U) | 0.5 |  |  |  |  |
| *NBN^185^* | F - 5´GGATGTAAACAGCCTCTTTG 3´  R - 5´CACAGCAACTATTACATCTC 3´ | 1x | 94ºC, 3min | MgCl_2_ (mM | 1.5 | 290 | *HinfI* | WT: 170; 120 | 65 |
|  |  | 35x | 94ºC, 45s  58ºC, 45s  72ºC,45s | primer (pmol) | 12.5 |  |  |  |  |
|  |  |  |  | DNA (ng) | 50 |  |  | H: 290; 170;120 |  |
|  |  |  |  | dNTPs (mM) | 0.25 |  |  |  |  |
|  |  | 1x | 72ºC, 5min | Buffer 10x Invitrogen^TM^ | 1x |  |  | HV:  290 |  |
|  |  |  |  | Taq DNA Polymerase (U) | 0.5 |  |  |  |  |
| *PARP1^762^* | F - 5´TTTGCTCCTCCAGGCCAACG 3´  R - 5´CATCGATGGGATCCTTGCTGCT 3´ | 1x | 95ºC, 5min | MgCl_2_ (mM) | 1.5 | 110 | *BstUI* | WT: 110 | 66 |
|  |  | 35x | 95º, 30s  55ºC, 35s  72ºC, 45s | primer (pmol) | 10 |  |  |  |  |
|  |  |  |  | DNA (ng) | 100 |  |  | H:  110; 90 |  |
|  |  |  |  | dNTPs (mM) | 0.25 |  |  |  |  |
|  |  |  |  | Buffer 10x Invitrogen^TM^ | 1x |  |  | HV: 90 |  |
|  |  | 1x | 72ºC, 10min | Taq DNA Polymerase (U) | 0.5 |  |  |  |  |

WT- wild-type; H - heterozygous variant; HV - homozygous variant; na – not applicable
